# Localization of an Underwater Control Network Based on Quasi-Stable Adjustment

**DOI:** 10.3390/s18040950

**Published:** 2018-03-23

**Authors:** Jianhu Zhao, Xinhua Chen, Hongmei Zhang, Jie Feng

**Affiliations:** 1 School of Geodesy and Geomatics, Wuhan University, 129 Luoyu Road, Wuhan 430079, China; jhzhao@sgg.whu.edu.cn (J.Z.); 2016102140023@whu.edu.cn (J.F.); 2 Institute of Marine Science and Technology, Wuhan University, Wuhan 430079, China; 3 Automation Department, School of Power and Mechanical Engineering, Wuhan University, Wuhan 430072, China; hmzhang@whu.edu.cn

**Keywords:** underwater control network, acoustic ranging, quasi-stable adjustment, baseline quality control, depth constraint

## Abstract

There exists a common problem in the localization of underwater control networks that the precision of the absolute coordinates of known points obtained by marine absolute measurement is poor, and it seriously affects the precision of the whole network in traditional constraint adjustment. Therefore, considering that the precision of underwater baselines is good, we use it to carry out quasi-stable adjustment to amend known points before constraint adjustment so that the points fit the network shape better. In addition, we add unconstrained adjustment for quality control of underwater baselines, the observations of quasi-stable adjustment and constrained adjustment, to eliminate the unqualified baselines and improve the results’ accuracy of the two adjustments. Finally, the modified method is applied to a practical LBL (Long Baseline) experiment and obtains a mean point location precision of 0.08 m, which improves by 38% compared with the traditional method.

## 1. Introduction

Underwater control networks are widely applied to underwater navigation, seafloor crustal dynamics observation and stability monitoring of underwater structure through measuring the absolute coordinates of the monitored object [1,2]. To get the coordinates of the object, it is necessary to determine the absolute coordinates of all the control points in the underwater control network firstly.

The measurement to determine the absolute coordinates of the underwater control network can be divided into two types:

(1) Absolute measurement:

This kind of measurement can directly obtain the absolute coordinates of the underwater control point by using GNSS (Global Navigation Satellite System) technology and multiple sensors, also called absolute datum transfer.

The most popular way is the GPS-acoustic (Global Positioning System-acoustic) method [2]. It uses several survey vessels equipped with a transducer and GNSS to realize acoustic ranging rendezvous with underwater control points equipped with transponders [3,4]. During the measurement, GNSS precisely offers the absolute coordinates of the on-board transducer. On-board transducers constantly transmit the beam, and underwater transponders receive and respond to on-board transducers. However, it was found that the positioning precision is affected seriously by velocity errors by decreasing the precision of the distance from the on-board transducer to the seafloor acoustic transponder [5,6].

To improve the positioning precision, scholars tried different ways to deal with velocity errors. The most common method is to carry out CTD (sea water Conductivity, Temperature and Depth) observations to get an accurate acoustic velocity structure of the corresponding water area and then through the ray-tracing method to get the final slant distance [7,8,9,10,11,12]. However, the velocity varies with time and space, so it is practically impossible to get an accurate acoustic velocity structure for all the acoustic ranging [2]. Therefore, under this mode of measurement, in data post-processing, scholars use other treatments for the distance on the basis of ray-tracing. For example, Fanlin Yang et al. (2011) regarded remaining ranging error after ray-tracing as an unknown parameter to participate in the later calculation [13]. All these treatments improve the precision of the slant distance, but they increase the complexity of data processing.

Then, a new measurement mode was proposed called circle navigation in 2007, which largely decreases the impact of velocity errors on plane coordinates [14]. It uses one survey vessel around one unknown point for circular sailing. Relative to the GPS-acoustic method, the circle navigation incident angle of the measuring beam is approximately equal, as well as the ranging error brought by velocity, which can be effectively eliminated after the least squares solution [14,15]. However, when there are many points to be determined in an underwater control network, circle navigation becomes time consuming and laborious.

As for the vertical coordinate, there are specialized sensors recording seafloor pressure, gravity or magnetic data to determine the depth of the underwater control point with the progress of technology, the precision of which is better than that of the GPS-acoustic method and circle navigation [16,17,18,19]. These sensors are generally installed at underwater points and are used in relative measurement, as well.

(2) Relative measurement:

This kind of measurement is committed to obtaining the slant distance or azimuth of every two underwater points by the transponders’ mutual response.

Because underwater transponders on the two endpoints at the baseline have a small depth difference, the impact of velocity error on baseline observation is quite small relative to the absolute measurement [16,18]. Therefore, the precision of relative measurement is high [20,21]. However, relative measurement alone cannot obtain the absolute coordinates of the underwater control network when no absolute coordinates of any point are known.

Due to the advantages and disadvantages of the two kinds of measurement, in recent years, it has become popular to combine absolute measurement with the relative one [19,20,21,22,23], such as the Sonardyne (Sonardyne, Hampshire, UK) 6G LBL (Long Baseline) system. Firstly, the on-board transducer continuously transmits the command signal to the underwater transponder during the voyage of the ship. Once the transponder receives the signal, it will be activated and telemeters data to the transducer. The survey is actually the absolute measurement, and it is to obtain the absolute 3D coordinates of several underwater points in the control network, mostly by circle navigation [14,15]. After the absolute measurement, underwater transponders start another mode of work. They communicate with each other in the same way as thee transducer and transponder to obtain the slant distance of every two underwater points. Compared with the previous underwater positing system, the new system adopted a wider bandwidth and more channels to guarantee stable and accurate positioning signals, far propagation distance and more user applications. During the whole measurement, pressure sensors work continuously, obtaining the depth sequence of each point. Because both circle navigation and the mutual response of underwater transponders are based on TWTT (Two-Way Travel Time) [15,18], CTD observations are carried out in the survey area, and all the distances are obtained through ray-tracing.

After data collection finishes, it is necessary to process the data to get the absolute coordinates of unknown points in the network. The current method is to carry out the least square method [20], in which the coordinates obtained by absolute measurement are regarded as starting data, the distances obtained by the relative one as observations and the depths obtained by pressure sensors as observations or known values. When the depths are regarded as observations, the method is called 3D Constraint Adjustment with depth constraint (3DCA) [22]. When the depths are regarded as known values, it is 2D Constraint Adjustment with depth constraint (2DCA) [22].

However, the positioning precision of absolute measurement is not high, just the decimeter-level, while the baseline precision in relative measurement is high, up to the millimeter-level [14,17], which is much higher than positioning precision. It would cause the precise network shape made up of underwater baselines to be destroyed to some extent after the introduction of imprecise known points in 3DCA or 2DCA.

Therefore, we propose to carry out quasi-stable adjustment for absolute coordinates of known points with underwater baselines as observations before constraint adjustment, to amend known points and increase the compatibility between known points and network shape. In addition, relative measurement can obtain plenty of repeating baselines to participate in quasi-stable adjustment and constraint adjustment, part of which may be polluted by the marine environment, signal leap of underwater transponders, acoustic wave multi-reflection, etc. To improve baseline accuracy and the inner coincidence precision of the two adjustments, we add unconstrained adjustment for baselines to eliminate the unqualified baselines. The structure of this paper is as follows. Section 2 elaborates 2D unconstrained adjustment as the quality control of underwater baselines. Section 3 describes 2D quasi-stable adjustment in detail. Section 4 presents the validation and analysis of the proposed method through experiments. Section 5 provides the corresponding discussion. Lastly, Section 6 presents several beneficial conclusions and recommendations that are drawn from the experiments and discussions.

## 2. Quality Control of Underwater Baselines

Before quasi-stable and constraint adjustment, it is necessary to control the quality of the observations, that is underwater baselines from relative measurement. An unconstrained adjustment is proposed as follows.

During measurement, pressure sensors provide depth information of each underwater transponder. Imitating 2DCA and 3DCA, the introduction of the depth information in unconstrained adjustment can be divided into two types:

(1) Use the depth information to get the depth difference of every two underwater points, and further switch the slant distance into horizontal distance. Then, carry out 2D unconstrained adjustment.
(2) Both the depth difference and slant distance are regarded as observations. Then, carry out 3D unconstrained adjustment.

In 3D adjustment, if the value difference of one dimension is far less than the other two dimensions, the result of the adjustment is likely to be wrong [24]. For an underwater control network, it is common that the depth difference between underwater control points is rather small relative to the *x* and *y* difference. In this case, 3D unconstrained adjustment is inapplicable.

Because unconstrained adjustment is mainly to eliminate unqualified baselines and evaluate internal coincidence precision and the calculation process of 2D unconstrained adjustment is simpler than that of the 3D, here we choose 2D unconstrained adjustment to realize quality control.

Assuming that *i* and *j* are two underwater control points and $l_{ij}$ and $z_{ij}$ are observed slant distance and depth difference between the two, so the plane distance $s_{ij}$ is:

(1) $$s_{ij}\;=\;\sqrt{l_{ij}^{2}\;-\;z_{ij}^{2}}$$

Meanwhile, supposing that $X_{i}(x_{i},y_{i})$ and $X_{j}(x_{j},y_{j})$ are real 2D coordinates of points *i* and *j* respectively, $g(X_{i},X_{j})$ represents the real plane distance of two points and Δs is the observation error, then the plane distance $s_{ij}$ can be represented by Equation (2) as follows:

(2) $$s_{ij}\;=\;g(X_{i},X_{j})\;+\;\Delta s\;=\;\sqrt{(x_{i}\;-\;x_{j}{)}^{2}\;+\;{\left(y_{i}\;-\;y_{j}\right)}^{2}}\;+\;\Delta s$$

Taylor expand Formula (2), then the correction of the underwater baseline is:

(3) $$\begin{array}{l} v_{ij}\;=\;\frac{1}{s_{ij}^{0}}((x_{i}^{0}\;-\;x_{j}^{0})(d_{x_{i}}\;-\;d_{x_{j}})\;+\;(y_{i}^{0}\;-\;y_{j}^{0})(d_{y_{i}}\;-\;d_{y_{j}}))\;-\;(s_{ij}^{}\;-\;s_{ij}^{0})\\ =\;[\frac{x_{i}^{0}\;-\;x_{j}^{0}}{s_{ij}^{0}}\;\frac{y_{i}^{0}\;-\;y_{j}^{0}}{s_{ij}^{0}}\;-\;\frac{x_{i}^{0}\;-\;x_{j}^{0}}{s_{ij}^{0}}\;-\;\frac{y_{i}^{0}\;-\;y_{j}^{0}}{s_{ij}^{0}}]\left[\begin{array}{c} d_{x_{i}}\\ d_{y_{i}}\\ d_{x_{j}}\\ d_{y_{j}} \end{array}\right]\;-\;(s_{ij}^{}\;-\;s_{ij}^{0}) \end{array}$$

In the formula, $\left(x_{i}^{0},y_{i}^{0}\right)$ and $\left(x_{j}^{0},y_{j}^{0}\right)$ are a priori coordinates of points *i* and *j*, respectively. $\left(d_{x_{i}},d_{y_{i}}\right)$ and $\left(d_{x_{j}},d_{y_{j}}\right)$ are corrections of *i* and *j*, respectively. $s_{ij}^{0}$ is the a priori value of the underwater baseline, which is calculated by $\left(x_{i}^{0},y_{i}^{0}\right)$ and $\left(x_{j}^{0},y_{j}^{0}\right)$. $s_{ij}$ is the observed value, obtained by Equation (1).

Supposing there are *n* underwater baselines (including repeating baselines) and *m* underwater control points, then there are *n* equations, like Equation (3). Combine the *n* equations to build the correction equation group as Equation (4).

(4) $$\underset{n\times 1}{V}\;=\;\underset{n\times 2m}{B}\underset{2m\times 1}{\hat{x}}\;-\;\underset{n\times 1}{l}$$

In Equation (4), **V** is the collection of $v_{ij}$. **V** is the correction matrix of underwater baselines. **B** is the collection of $\left[\frac{x_{i}^{0}\;-\;x_{j}^{0}}{s_{ij}^{0}}\;\frac{y_{i}^{0}\;-\;y_{j}^{0}}{s_{ij}^{0}}\;-\frac{x_{i}^{0}\;-\;x_{j}^{0}}{s_{ij}^{0}}\;-\frac{y_{i}^{0}\;-\;y_{j}^{0}}{s_{ij}^{0}}\right]$. **B** is the coefficient matrix, and it is rank-deficient. $\hat{x}$ is the collection of $\left(d_{x_{i}},d_{y_{i}}\right)$ of all the underwater control points. $\hat{x}$ is the correction matrix of coordinates. **l** is the collection of $s_{ij}^{}-s_{ij}^{0}$.

Supposing normal matrix $\underset{2m\times 2m}{N}\;=\;\underset{2m\times n}{B^{T}}\underset{n\times n}{P}\underset{n\times 2m}{B}$, in which **P** is the weight matrix of underwater baselines, **N** is rank-deficient, as well. In matrix **P**, the weight of a baseline is generally set as the reciprocal of the length of the baseline [24,25]. In 2D unconstrained adjustment, it is the reciprocal of the plane distance.

Choose unconstrained adjustment with gravity datum, and the weight matrix **Px** of points is the unit matrix [24]. The datum matrix **S** is:

(5) $$S^{T}\;=\;[\begin{array}{c} 1\\ 0\\ -x_{1}^{0} \end{array}\begin{array}{c} 0\\ 1\\ y_{1}^{0} \end{array}\begin{array}{c} 1\\ 0\\ -x_{{}_{2}}^{0} \end{array}\begin{array}{c} 0\\ 1\\ y_{{}_{2}}^{0} \end{array}\ldots \begin{array}{c} 1\\ 0\\ -x_{{}_{m}}^{0} \end{array}\begin{array}{c} 0\\ 1\\ y_{{}_{m}}^{0} \end{array}].$$

Supposing $K\;=\;PxSS^{T}Px$, then the coefficient matrix of the normal equation is $N^{\prime }\;=\;N\;+\;K$ [24], and **N′** is full rank at the moment. The correction matrix of coordinates $\hat{x}$ is:

(6) $$\hat{x}\;=\;{(N^{\prime })}^{-1}B^{T}Pl$$

The unit weight mean square error $\sigma_{0}$ and the mean square error $\sigma_{ij}$ of each baseline are:

(7) $$\begin{array}{l} \sigma_{0}\;=\;\sqrt{\frac{V^{T}PV}{n\;-\;rank(B)}}\\ \sigma_{ij}\;=\;\sigma_{0}\;/\;\sqrt{P_{ij}} \end{array}$$

Among them, *rank*(**B**) represents the rank of coefficient matrix **B**. $P_{ij}$ is the weight of the corresponding underwater baseline.

Finally, use Equation (8) to eliminate disqualified baselines:

(8) $$\mathrm{If}\;\begin{array}{c} v_{ij}\;>\;k\sigma_{ij}\\ \mathrm{Otherwise}\; \end{array}\;\mathrm{then}\;\begin{array}{c} \mathrm{Refused}\\ \;\mathrm{Accepted} \end{array}$$

Inside, $v_{ij}$ is the correction of baselines. $\sigma_{ij}$ is the error of the corresponding baseline. *k* is the multiple in the threshold, which needs to be set appropriately. Generally, *k* is set as 3, 2 or 1 [25,26]. It also can be decimal [26]. After eliminating disqualified baselines, do unconstrained gravity datum adjustment until all the baselines are qualified.

**Figure 1:**
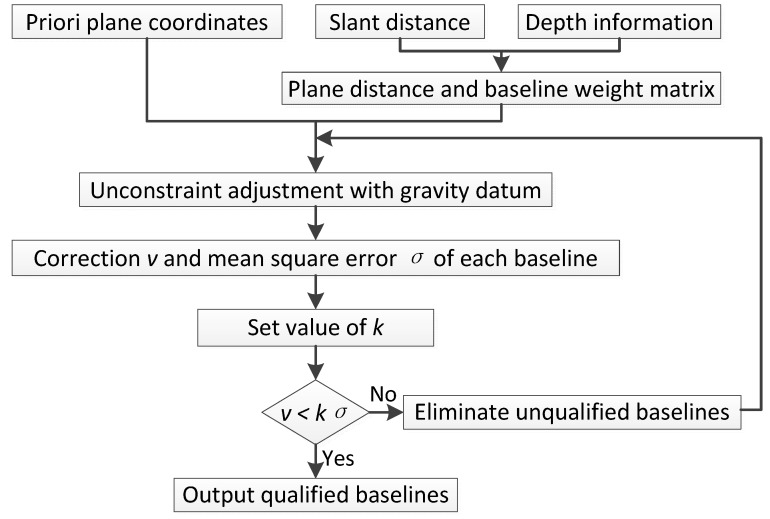
Data process of 2D unconstrained adjustment.

The whole process is depicted as follows (Figure 1).

After quality control of underwater baselines is finished, it is time to carry out quasi-stable adjustment.

## 3. Quasi-Stable Adjustment

To increase the compatibility of starting data and the underwater network, we add quasi-stable adjustment for known points obtained from absolute measurement to mend their coordinates, with regard to the qualified underwater baselines obtained by the above step as observations. Because the vertical coordinate can be obtained precisely by depth from the pressure sensor, wave sensor, tide sensor and water surface elevation [19], here we also just focus on the 2D coordinates of known points. The data process of 2D quasi-stable adjustment is as follows.

**Figure 2:**
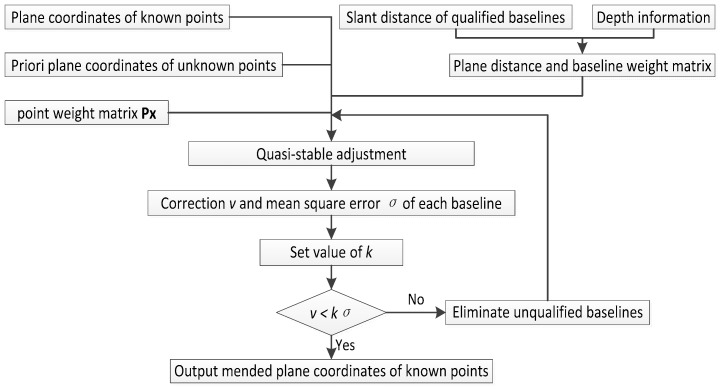
Data process of 2D quasi-stable adjustment.

In Figure 2, the plane distance of underwater baseline is obtained by Formula (1), and it also can be expressed by Formula (2). The baseline weight matrix is the same as **P** in unconstrained adjustment, in which the weight of a baseline is the reciprocal of the plane distance. The point weight matrix **Px** is a diagonal matrix, in which the weight of known points is o, and for the others is zero.

Then, Taylor expand Equation (2). When the two endpoints of the baselines are both unknown, the correction of the underwater baseline is expressed by Equation (3). Otherwise, replace the a priori coordinates of known points with their absolute coordinates obtained by absolute measurement.

Therefore, if there are *n* baselines, absolute coordinates of known points, a priori coordinates of unknown points and plane distance of underwater baselines form *n* correction equations like Equation (3). These equations make up equation group as Formula (4).

In quasi-stable adjustment, the correction and mean square error of points and baselines can be calculated by Equations (4)–(7). However, it needs to noted that the point weight matrix **Px** and datum matrix **S** of quasi-stable adjustment are different from unconstrained adjustment. The value of **Px** has been depicted above. In Equation (5), that is datum matrix **S**, the a priori coordinates of known points should be replaced with their absolute coordinates obtained by absolute measurement.

After quasi-stable adjustment has finished, the baselines will be further eliminated by Equation (8). When the iteration ends, the coordinates of known points have been fine-tuned. Finally, we regard the mended coordinates as the starting data to participate in constraint adjustment, 3DCA or 2DCA, to get unknown underwater points [22], with the qualified baselined obtained by unconstrained adjustment as observations.

**Figure 3:**
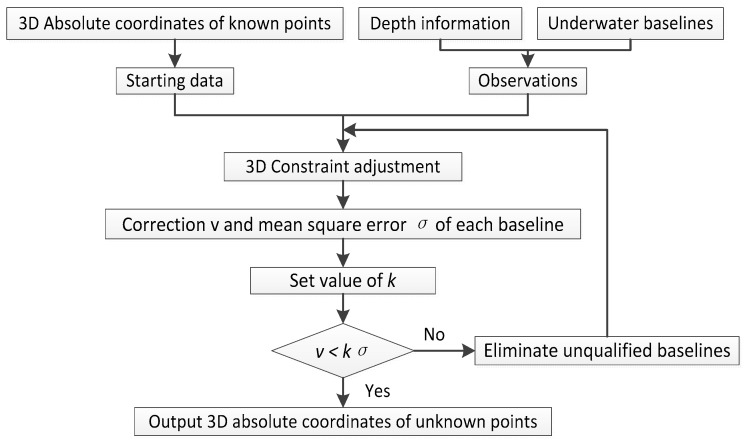
Data process of 3D Constraint Adjustment (3DCA).

**Figure 4:**
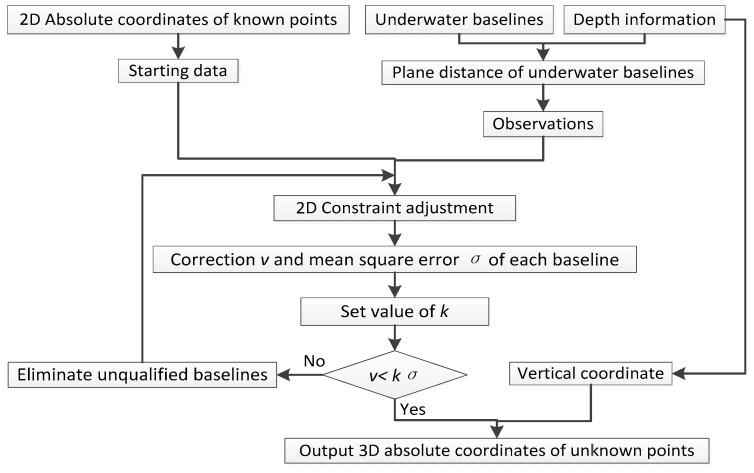
Data process of 2DCA.

For the sake of understanding the experiment below, we give the flowchart of 3DCA and 2DCA, as shown in Figure 3 and Figure 4.

## 4. Experiment and Analysis

After all the proposed methods have been depicted, they are applied to a practical LBL experiment, and the results are compared with that of other recent approaches. The experiment and comparisons are depicted as follows.

### 4.1. Data Acquisition

**Figure 5:**
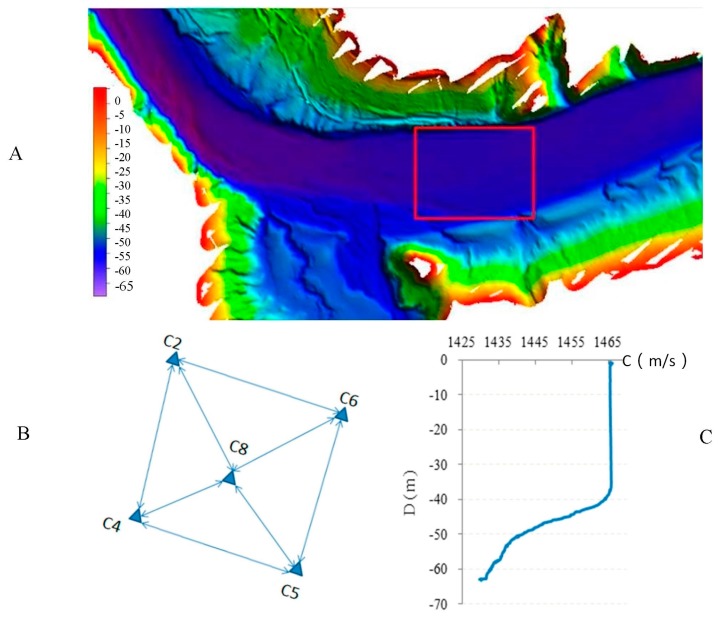
Underwater topography (**A**), control network (**B**) and sound velocity profile (**C**) in the experiment area. (**A**) The red box represents the survey area, the center of which is around 126.5° E, 43.4° N. The color from red to purple represents the depth from the minimum value to the maximum value. (**B**) Five small triangles represent underwater control points. (**C**) The horizontal axis represents sound velocity, and the ordinate axis represents depth.

In order to verify the correctness of the proposed method, experiments were carried out in Songhua Lake in September 2015, as shown in Figure 5A. The average depth of water in the experimental area is about 60 m. The ups and downs of underwater topography change at 1–2 m, and the change is relatively flat, as shown in the red box in Figure 5A.

In the experiment, the positioning equipment adopted the Sonardyne 6G LBL system, including 6 elements and their floating bodies, 1 tracking and positioning transceivers (including telemetry transducer) and deck units. The ship attitude sensor adopted was Seatex MRU-05 (Kongsberg Seatex AS, Trondheim, Norway), and the compass adopted was TSS Meridian Surveyor (TELEDYNE TSS, Hertfordshire, UK), which were mainly used for measuring ship attitudes and azimuth during the measurement. The sound velocity profiler adopted was AML SVP (AML Oceanographic, BC, Canada), and bathymetry adopted was ValePort 740 (Valeport, Devon, United Kingdom). The survey ship used Leica GPS1200 (Leica Geosystems AG, Heerbrugg, Switzerland) to locate with the aid of GNSS RTK (Real-Time Kinematic) technology. The list of performance parameters for the above device is shown in Table 1. In the default accuracy column in Table 1, *S* is observed acoustic ranging distance and *D* is observed depth.

At the bottom of the lake, we set 5 transponders as the underwater control points in a 134 m × 102 m range, as shown in Figure 5B, numbered C2, C4, C5, C6 and C8. Before measuring, we strictly determined the coordinates of ship-borne transducer and the GNSS RTK antenna in the coordinate system of the ship. When measuring, we first carried out circle navigation on 5 underwater control points respectively for 4 laps with a radius of 60 m. Then, we carried out mutual acoustic ranging between underwater points (transponders), and several observation sets were completed. During the measurement, we also carried out the measurement of the sound velocity profile in the experiment area, as shown in Figure 5C.

### 4.2. Data Processing and Analysis

After data acquisition, data processing of circle navigation, unconstrained, quasi-stable and constraint adjustment of the underwater control network were carried out, and the absolute coordinates of all the underwater control points were obtained. The following describes the data processing in detail.

(1) Circle navigation:

Circle navigation obtained the absolute 3D coordinates of the on-board transducer and the distance from the on-board transducer to the underwater transponder. We carried out the least squares solution to realize acoustic ranging rendezvous and combined the depth information of pressure sensors to get the absolute coordinates and internal coincidence accuracy of the five underwater control points, as Table 2.

In the table, *x*, *y*, *z* are the absolute coordinates and $\sigma_{x}$, $\sigma_{y}$, $\sigma_{z}$ are the corresponding accuracies. σ is the internal coincidence accuracy of the point, which was calculated by:

$$\sigma \;=\;\sqrt{\left(\sigma_{x}^{2}\;+\;\sigma_{y}^{2}\;+\;\sigma_{z}^{2}\right)}$$

(2) Unconstrained adjustment:

Two thousand six hundred twenty six underwater baselines were obtained by relative measurement, including repeating baselines. Using the depth difference of the five control points obtained by pressure sensors, we converted the observed slant distance to horizontal distance according to Formula (1) and constructed observation equations like Formulas (3) and (4). Then, we used the 2D unconstrained method of Section 2 to process data and rejected unqualified baselines according to Formula (8), in which we set the multiple k as 2. We finally obtained 1512 qualified baselines.

**Figure 6:**
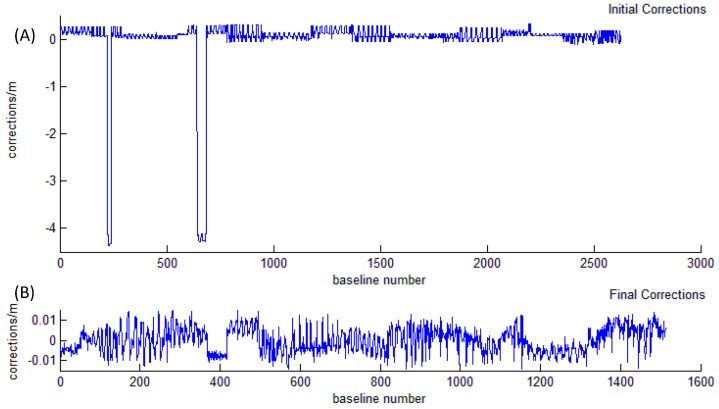
Baseline corrections of unconstrained adjustment. (**A**) denotes the initial corrections of baselines before quality control; (**B**) denotes the final corrections of baselines after quality control.

Figure 6 shows the effect of quality control for underwater baselines. In Figure 6A, the maximal correction is up to about 4.5 m, which can be considered as an error. Except for a few outliers, others points are within 0.5 m. In Figure 6B, all the baseline corrections are less than 0.015 m, indicating that the final internal coincidence accuracy is much higher than before, and the underwater baselines have obtained valid quality control.

(3) Quasi-stable adjustment and constraint adjustment:

In practical measurement, the fewer the points of circle navigation, the greater the savings. The most economical way is to measure a certain number of points, which can just meet the necessary starting data of constraint adjustment. Here, the number is 2. In the next data processing, it is supposed that we just obtained 2 known points from circle navigation. Then, there are 10 ($C_{5}^{2}$) combinations.

For the 10 combinations, we carried out the following 4 methods with the qualified baselines as observations to obtain the absolute coordinates of the remaining unknown underwater control points. Between the four, 2DCA and 3DCA are recent approaches and 2D Quasi-stable Constraint Adjustment (2DQCA) and 3DQCA are the proposed methods. In the four methods, the value of k was set as 3.

(1) 2DCA (2D Constraint Adjustment with depth constraint);
(2) 3DCA(3D Constraint Adjustment with depth constraint);
(3) Quasi-stable adjustment and then 2DCA (2DQCA);
(4) Quasi-stable adjustment and then 3DCA (3DQCA).

Finally, each method obtained 10 combinations, in total, 40 (4 × $C_{5}^{2}$) sets of results. Taking the absolute coordinates in Table 2 as the reference, respectively calculate the 3D coordinate difference of the unknown points, and regard the difference as the external coincidence precision, as shown in Table 3.

In Table 3, *dx*, *dy*, *dz* represent the external coincidence precision in the *x*, *y*, *z* direction, respectively. Their units are all meters.

It can be seen from Table 3 that:

(1) In the same combination, the most absolute value of *dx* and *dy* of 2DQCA and 3DQCA is smaller than that of 2DCA and 3DCA. Especially in some combinations, such as ‘C2 C4′ and ‘C6 C8′, *dx* and *dy* in the two traditional methods have decimeter-level values while, in the two new methods, the values are all within the centimeter level.
(2) The value of *dz* is near the four methods.
(3) The external coincidence precision in the *x*, *y*, *z* direction is similar for 2DQCA and 3DQCA. This phenomenon also existed for 2DCA and 3DCA.
(4) The value of *dz* is the same for 2DCA and 2DQCA. That is because the means of the acquisition of *z* of 2DCA and 2DQCA is the same, which only depends on pressure sensors.

Table 3 shows that in most combinations, the external coincidence precision of the new methods is higher than the traditional ones, especially for plane coordinate precision. The precision is similar for the two new methods, as well as for the two traditional ones.

**Figure 7:**
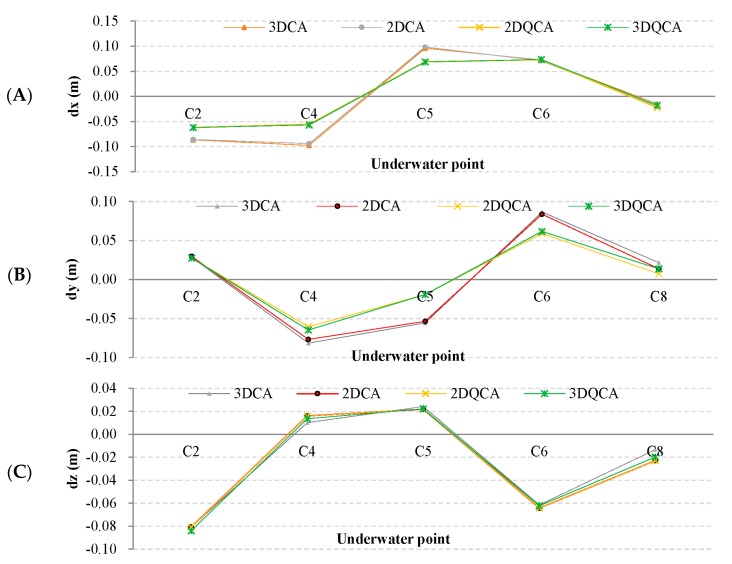
The external coincidence precision of the 4 methods in *x*, *y*, *z* direction, respectively. The green, red, purple and yellow lines represent 3DCA, 2DCA, 2DQCA and 3DQCA, respectively. (**A**) represents mean *dx* of each point; (**B**) represents mean *dy* of each point; (**C**) represents mean *dz* of each point.

For a more visual comparison, we respectively averaged *dx*, *dy*, *dz* of each unknown point for each method and draw a line diagram as shown in Figure 7.

In Figure 7A,B, the lines of 2DCA and 3DCA almost coincide, as well as 2DQCA and 3DQCA. The lines of new methods are nearer to the horizontal axis, which means the external coincidence precision of the new methods is higher than the traditional for *x* and *y* direction. This is because quasi-stable adjustment increases the compatibility of the known points’ plane coordinates with the underwater control network shape.

In Figure 7C, the trends of four lines are similar, which reflects that the external coincidence precision of the 4 methods is similar for the vertical direction. Among them, the lines of 2DCA and 2DQCA coincide completely. The reason has been explained above.

Finally, we calculated plane precision $p_{\mathrm{plane}}$ and positional precision $p_{\mathrm{point}}$ of each unknown point by the following formula:

$$\begin{array}{l} p_{\mathrm{plane}}\;=\;\sqrt{dx^{2}\;+\;dy^{2}}\\ p_{\mathrm{point}}\;=\;\sqrt{dx^{2}\;+\;dy^{2}\;+\;dz^{2}} \end{array}$$

Then, average the plane and positional precision of the unknown points in the four methods respectively to get their average plane and positional precision. For example, 2DCA got 30 (3 × $C_{5}^{2}$) unknown points in total (includes repeating points, that is, all combinations of 2DCA obtained 6 sets of coordinates for each point), we first calculated the plane and positional precision of each point and then averaged them. We also calculated other statistical data, shown in Table 4.

It can be seen that the average plane and positional precision of 2DCA and 3DCA are almost same, as well as 2DQCA and 3DQCA. The precision of the new methods is higher than that of the traditional ones. As for the standard deviation of plane and positional precision, the value of 2DQCA and 3DQCA is smaller than that of 2DCA and 3DCA, which means the new methods have better robustness.

## 5. Discussion

It has been verified that the precision and the robustness of the proposed methods are better than those of the traditional by the above experiments. In the following, some details about the unconstrained adjustment are discussed. The reason why the positional precision of 2DQCA and 3DQCA is close was analyzed.

### 5.1. The Significance of Unconstrained Adjustment

In the experiment, the quality of observed baselines in quasi-stable and constraint adjustment has been controlled by unconstrained adjustment. However, quasi-stable and constraint adjustment also have steps for baseline quality control, like Equation (7).

To verify the necessity of unconstrained adjustment, we chose C2 and C4 as known points to carry out the 2DQCA without unconstrained adjustment. The results were compared with those of normal 2DQCA with the same known points, shown in Table 5.

In Table 5, the external coincidence precision of the *x*, *y* direction and positional precision of 2DCQA are higher than those of 2DQCA without unconstrained adjustment, which shows that unconstrained adjustment is indispensable in the localization of the underwater control network.

In method 2DQCA, constraint adjustment only eliminated three baselines, and in 2DQCA without unconstrained adjustment, constraint adjustment only eliminated 53 baselines, while unconstrained adjustment eliminated 1114 baselines. Therefore, quality control is mainly dependent on unconstrained adjustment.

### 5.2. Setting of the k Value

**Figure 8:**
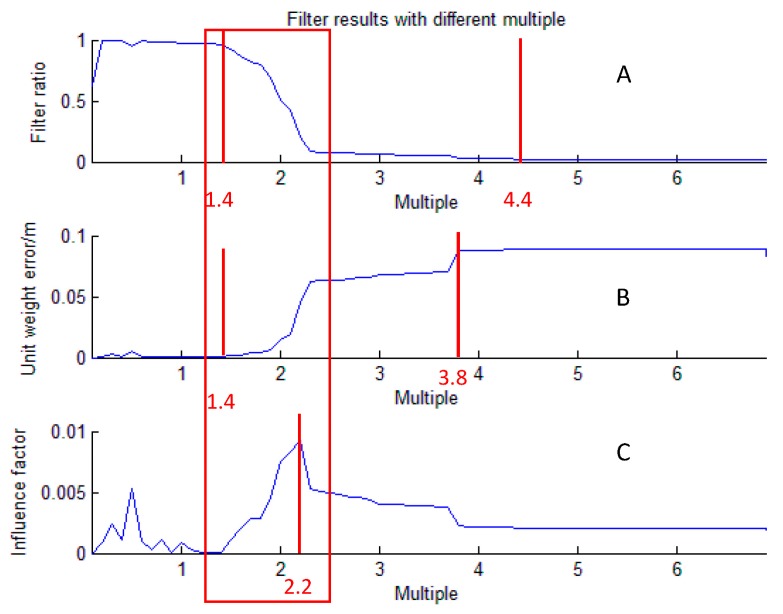
The change of filter ratio (**A**), unit weight error (**B**) and influence factor (**C**) with different multiple *k.* The red lines represent the change points, and the numbers below the lines represent the corresponding k value of the points. The area in the red box has the largest change in the curve. (**A**) The abscissa axis represents the value of *k*, and the vertical axis represents the filter ratio. (**B**) The abscissa axis represents the value of *k*, and the vertical axis represents the unit weight error of the underwater network. (**C**) The abscissa axis represents the value of *k*, and the vertical axis represents the influence factor, which equals the filter ratio multiplied by the unit weight error.

Since unconstrained adjustment is necessary, then the quality control threshold should be appropriately set. When we tried different multiple *k* in Equation (8), we found that the change of the filter ratio (the percentage of eliminated baselines in all the baselines) and the unit weight error of the underwater network had a trend, as shown in Figure 8.

It can be seen from Figure 8A,B that:

(1) In Figure 8A, when *k* < 1.4, the filter ratio is almost up to 100%. When 1.4 < *k* < 4.4, with the increase of multiple *k*, the filter ratio decreases. When *k* > 4.4, filter ratio tends to be constant, near 0%.
(2) In Figure 8B, when *k* < 1.4, the unit weight error is almost down to zero. When 1.4 < *k* < 3.8, the unit weight error increases with the increase of *k*. When *k* > 3.8, the unit weight error tends to be constant, near 0.1 m.

According to Figure 8A,B, we can find two points:

(1) The filter ratio reflects the number of eliminated baselines. When *k* < 1.4, almost all the baselines were eliminated, and when *k* > 4.4, almost no baselines were eliminated. These two cases are inadvisable. To obtain enough and reliable baselines, we should choose a *k* value between 1.4 and 4.4.
(2) The unit weight error reflects the inner coincidence accuracy of the underwater control network. The smaller the error is, the better the localization accuracy is. To obtain good inner coincidence accuracy, we should make the *k* value as small as possible.

When *k* < 1.4, the unit weight error was almost down to zero, but almost all the baselines were eliminated, which would lead to low reliability. Therefore, we should find a balance between unit the weight error and filter ratio, that is we require that baselines have not only high reliability, but also good inner coincidence accuracy.

To consider both factors above and try to find a suitable *k*, we tried to cross and magnify the influence of the two factors, and obtained the formula below.

influence factor = filter ratio × unit weight error

The higher the influence factor is, the more appropriate *k* is. We defined the influence factor as judgment criteria, and drew its change with the increase of *k*, as shown in Figure 8C.

It can be seen in Figure 8C that when *k* = 2.2, the influence factor is the peak. Because *k* is generally 3, 2 or 1 [25,26], we chose two as the value of *k* in the experiment.

In practical applications, we can just calculate the influence factor when *k* = 3, 2 or 1 and select the *k* that has the highest influence factor as the final value.

### 5.3. The Reason for the Close Positional Precision of 2DQCA and 3DQCA

In Table 3 and Table 4, we found that the positional precision of 2DQCA and 3DQCA is close, which is the same for 2DCA and 3DCA.

The difference of 2DQCA and 3DQCA is that 2DQCA takes 2DCA as the constraint adjustment, while 3DQCA takes 3DCA as the constraint adjustment. Therefore, the reason why the positional precision of 2DQCA and 3DQCA is close is the same reason why the positional precision of 2DCA and 3DCA is close. We analyze 2DCA and 3DCA below.

3DCA regards depth as observations with error and combines it and baselines to form the error equation group [22]. 2DCA considers the error of depth to be zero and uses it to convert slant distance to plane distance, as Equation (1) [22].

Using the total differentiation method for Equation (1), we obtain the plane distance error *ds*:

(9) $$ds\;=\;\frac{l}{s^{0}}dl\;-\;\frac{z}{s^{0}}dz，s^{0}\;=\;\sqrt{l^{2}\;-\;z^{2}}$$

In the formula, *l* and *z* are the observed slant distance and depth difference between the two points. *dl* and *dz* are the errors of *l* and *z*. $s^{0}$ is the corresponding plane distance.

Supposing *l* and *z* are not related, make $G\;=\;\left[\begin{array}{cc} \frac{l}{s^{0}} & \frac{-z}{s^{0}} \end{array}\right]，\sigma^{2}\;=\;\left[\begin{array}{cc} \sigma_{l}^{2} & 0\\ 0 & \sigma_{z}^{2} \end{array}\right]$. $\sigma_{l}$, $\sigma_{z}$ are the mean square errors of slant distance and depth difference, respectively. Then, the variance of plane distance is:

(10) $$\begin{array}{l} \sigma_{s}^{2}\;=\;G\sigma^{2}G^{T}\;=\;{\left(\frac{l}{s^{0}}\right)}^{2}\sigma_{l}^{2}\;+\;{\left(\frac{z}{s^{0}}\right)}^{2}\sigma_{z}^{2}\;=\;\frac{{\left(s^{0}\right)}^{2}\;+\;z^{2}}{{\left(s^{0}\right)}^{2}}\sigma_{l}^{2}\;+\;{\left(\frac{z}{s^{0}}\right)}^{2}\sigma_{z}^{2}\\ =\;\sigma_{l}^{2}\;+\;{\left(\frac{z}{s^{0}}\right)}^{2}(\sigma_{l}^{2}\;+\;\sigma_{z}^{2}) \end{array}$$

In Equation (10), $\sigma_{s}$ is the baseline error of 2DCA, which is also the observation error. $\sigma_{l}$ is the baseline error of 3DCA. ${\left(\frac{z}{s^{0}}\right)}^{2}(\sigma_{l}^{2}\;+\;\sigma_{z}^{2})$ is the naturalization error.

In Table 2, it can be seen that the depth difference *z* among the five points is small, 0.438 m on average, while plane distance is not small, 102.083 m on average. Then, in Equation (10), the average of ${\left(\frac{z}{s^{0}}\right)}^{2}$ can be 1.8 × 10^−5^. In addition, it can be seen from the previous content that $\sigma_{l}$ and $\sigma_{z}$ are just centimeter-level. Therefore, the naturalization error in the experiment is so small that it can be thought that $\sigma_{s}^{2}\;\approx \;\sigma_{l}^{2}$.

2DCA distributes baseline error $\sigma_{s}$ and plane coordinates error of known points to plane coordinates of unknown points, while 3DCA distributes baseline error $\sigma_{l}$, depth error $\sigma_{z}$ and 3D coordinates error of known points to 3D coordinates of unknown points. Because pressure sensors have high precision, depth error $\sigma_{z}$ is small. Therefore, the errors in 2DCA and 3DCA are approximately equal, and the positional precision of 2DCA and 3DCA is close, which is the same for 2DQCA and 3DQCA.

## 6. Conclusions and Suggestions

The proposed method, localization of underwater control network based on quasi-stable adjustment, realizes the high-accuracy positioning of underwater control points. Compared with the traditional positioning methods, such as 2D Constraint Adjustment (2DCA) and 3D Constraint Adjustment (3DCA), the proposed method has good performances in high-precision and stable solutions and low restriction for the precision of known points. These conclusions have been verified by experiments. In Songhua Lake, the proposed method eliminated 42% of baselines and ensured the baseline correction within 0.015 m in the unconstrained adjustment, as well as achieved mean positional precision of 0.08 m, which improved by 38% compared with the traditional adjustment (2DCA or 3DCA).

The experiment data were obtained in Songhua Lake, which had a calm water surface during the experiment. Therefore, the depth data provided by pressure sensors are regarded as known in the proposed 2DQCA. If the measurement is carried out in the sea, it is recommended that the tidal effect be considered to ensure the acquisition of accurate depth.

The proposed method will play a great role when the coordinates of known points in underwater control networks are not precise enough. However, the proposed method was just tested in a lake. Therefore, we are going to carry out the further experiments in deeper water. Furthermore, the reason for the low precision of absolute measurement is that the error of baselines from the transducer to the transponder is large. Therefore, we will also focus on how to reduce the baseline error in the next work.

## Figures and Tables

**Table 1:** Performance of the equipment.

Equipment	Measurement	Default Accuracy
Sonardyne 6G LBL	Ranging	±0.1% *S*
ValePort 740	Depth	±0.1% *D*
Seatex MRU-05	Roll, pitch, heave	±0.05°, ±0.05°, ±5 cm
TSS Meridian Surveyor	Heading	±0.1°
AML SVP	Sound velocity	±0.1 m/s
Leica GPS1200 RTK	Horizontal positioning	±0.03 m
	Vertical positioning	±0.05 m

**Table 2:** The absolute coordinates and accuracies of the five points by circle navigation.

Point	*x*/m	*y*/m	*z*/m	*σ_x_*/m	*σ_y_*/m	*σ_z_*/m	*σ*/m
C2	315,690.393	4,841,955.122	−40.88	0.017	0.017	0.006	0.025
C4	315,697.791	4,841,835.080	−40.13	0.011	0.011	0.005	0.016
C5	315,792.601	4,841,864.999	−40.02	0.017	0.016	0.008	0.025
C6	315,787.073	4,841,969.796	−40.6	0.017	0.016	0.007	0.024
C8	315,736.475	4,841,896.675	−40.44	0.018	0.018	0.007	0.026

**Table 3:** The result of 2DCA, 3DCA, 2D Quasi-stable Constraint Adjustment (2DQCA) and 3DQCA methods.

Method	Known Points	C2/m			C4/m			C5/m			C6/m			C8/m		
		*dx*	*dy*	*dz*	*dx*	*dy*	*dz*	*dx*	*dy*	*dz*	*dx*	*dy*	*dz*	*dx*	*dy*	*dz*
2DCA	C2 C4	/	/	/	/	/	/	0.155	−0.005	0.022	0.146	0.094	−0.060	0.084	0.039	−0.023
	C2 C5	/	/	/	−0.091	−0.051	0.016	/	/	/	0.110	0.046	−0.060	−0.063	−0.008	−0.023
	C2 C6	/	/	/	−0.079	−0.112	0.016	0.064	−0.087	0.022	/	/	/	−0.054	−0.099	−0.023
	C2 C8	/	/	/	−0.049	−0.089	0.016	0.084	−0.078	0.022	0.149	0.010	−0.060	/	/	/
	C4 C5	−0.056	0.115	−0.084	/	/	/	/	/	/	0.108	0.110	−0.060	−0.032	0.109	−0.023
	C4 C6	−0.117	0.019	−0.084	/	/	/	0.099	−0.030	0.022	/	/	/	−0.001	0.021	−0.023
	C4 C8	−0.001	0.077	−0.084	/	/	/	0.127	0.013	0.022	0.141	0.091	−0.060	/	/	/
	C5 C6	−0.158	−0.012	−0.084	−0.114	−0.117	0.016	/	/	/	/	/	/	−0.115	−0.020	−0.023
	C5 C8	−0.112	0.033	−0.084	−0.118	−0.050	0.016	/	/	/	0.030	0.086	−0.060	/	/	/
	C6 C8	−0.135	−0.063	−0.084	−0.053	−0.139	0.016	0.060	−0.074	0.022	/	/	/	/	/	/
3DCA	C2 C4	/	/	/	/	/	/	0.155	−0.004	0.019	0.146	0.095	−0.061	0.084	0.039	−0.013
	C2 C5	/	/	/	−0.099	−0.076	0.016	/	/	/	0.120	0.065	−0.060	−0.048	0.012	−0.023
	C2 C6	/	/	/	−0.096	−0.119	0.013	0.052	−0.098	0.019	/	/	/	−0.034	−0.068	−0.014
	C2 C8	/	/	/	−0.049	−0.088	0.014	0.083	−0.077	0.025	0.148	0.011	−0.063	/	/	/
	C4 C5	−0.056	0.115	−0.087	/	/	/	/	/	/	0.108	0.110	−0.061	−0.032	0.109	−0.012
	C4 C6	−0.117	0.019	−0.074	/	/	/	0.099	−0.030	0.034	/	/	/	−0.002	0.021	−0.001
	C4 C8	−0.002	0.077	−0.081	/	/	/	0.127	0.013	0.025	0.141	0.091	−0.058	/	/	/
	C5 C6	−0.158	−0.012	−0.086	−0.114	−0.117	0.013	/	/	/	/	/	/	−0.115	−0.021	−0.015
	C5 C8	−0.112	0.033	−0.080	−0.118	−0.049	0.014	/	/	/	0.031	0.086	−0.063	/	/	/
	C6 C8	−0.135	−0.063	−0.083	−0.053	−0.139	0.020	0.060	−0.073	0.023	/	/	/	/	/	/
2DQCA	C2 C4	/	/	/	/	/	/	0.061	−0.029	0.022	0.071	0.057	−0.060	−0.024	0.007	−0.023
	C2 C5	/	/	/	−0.057	−0.057	0.016	/	/	/	0.065	0.045	−0.060	−0.041	−0.017	−0.023
	C2 C6	/	/	/	−0.036	−0.065	0.016	0.082	−0.011	0.022	/	/	/	−0.016	0.008	−0.023
	C2 C8	/	/	/	−0.047	−0.073	0.016	0.074	−0.011	0.022	0.069	0.074	−0.060	/	/	/
	C4 C5	−0.075	0.015	−0.084	/	/	/	/	/	/	0.070	0.058	−0.060	−0.023	0.007	−0.023
	C4 C6	−0.057	0.023	−0.084	/	/	/	0.064	−0.021	0.022	/	/	/	−0.018	0.014	−0.023
	C4 C8	−0.074	0.017	−0.084	/	/	/	0.062	−0.026	0.022	0.072	0.061	−0.060	/	/	/
	C5 C6	−0.054	0.041	−0.084	−0.068	−0.050	0.016	/	/	/	/	/	/	−0.012	0.025	−0.023
	C5 C8	−0.057	0.034	−0.084	−0.067	−0.060	0.016	/	/	/	0.090	0.059	−0.060	/	/	/
	C6 C8	−0.056	0.036	−0.084	−0.059	−0.058	0.016	0.070	−0.017	0.022	/	/	/	/	/	/
3DQCA	C2 C4	/	/	/	/	/	/	0.061	−0.029	0.022	0.071	0.057	−0.062	−0.025	0.008	−0.019
	C2 C5	/	/	/	−0.061	−0.078	0.016	/	/	/	0.071	0.061	−0.060	−0.022	0.008	−0.023
	C2 C6	/	/	/	−0.042	−0.070	0.013	0.079	−0.013	0.023	/	/	/	−0.008	0.021	−0.019
	C2 C8	/	/	/	−0.047	−0.073	0.013	0.074	−0.011	0.023	0.068	0.074	−0.062	/	/	/
	C4 C5	−0.075	0.015	−0.084	/	/	/	/	/	/	0.070	0.058	−0.062	−0.023	0.006	−0.019
	C4 C6	−0.057	0.023	−0.085	/	/	/	0.064	−0.021	0.021	/	/	/	−0.018	0.014	−0.020
	C4 C8	−0.074	0.017	−0.084	/	/	/	0.062	−0.026	0.022	0.071	0.061	−0.063	/	/	/
	C5 C6	−0.055	0.041	−0.083	−0.068	−0.051	0.013	/	/	/	/	/	/	−0.012	0.025	−0.019
	C5 C8	−0.057	0.034	−0.084	−0.067	−0.060	0.013	/	/	/	0.091	0.059	−0.062	/	/	/
	C6 C8	−0.056	0.036	−0.084	−0.059	−0.058	0.012	0.070	−0.016	0.022	/	/	/	/	/	/

**Table 4:** The statistical data of the four methods.

	2DCA	3DCA	2DQCA	3DQCA
average plane precision/m	0.120	0.120	0.069	0.070
maximal plane precision/m	0.174	0.174	0.108	0.108
minimal plane precision/m	0.021	0.021	0.018	0.022
standard deviation of plane precision/m	0.033	0.035	0.024	0.025
average positional precision/m	0.130	0.129	0.084	0.083
maximal positional precision/m	0.184	0.184	0.125	0.124
minimal positional precision/m	0.031	0.021	0.030	0.029
standard deviation of positional precision/m	0.034	0.038	0.031	0.029

**Table 5:** The impact of unconstrained adjustment on 2DQCA.

	External Coincidence Precision	C5	C6	C8
2DQCA	*dx*/m	0.061	0.071	−0.024
	*dy*/m	−0.029	0.057	0.007
	*dz*/m	−0.022	0.060	0.023
	positional/m	0.071	0.109	0.034
2DQCA without unconstrained adjustment	*dx*/m	0.134	0.160	0.055
	*dy*/m	−0.018	0.070	0.032
	*dz*/m	−0.022	0.060	0.023
	positional/m	0.137	0.184	0.068
